# Generating dynamic carbon-dioxide traces from respiration-belt recordings: Feasibility using neural networks and application in functional magnetic resonance imaging

**DOI:** 10.3389/fnimg.2023.1119539

**Published:** 2023-02-16

**Authors:** Vismay Agrawal, Xiaole Z. Zhong, J. Jean Chen

**Affiliations:** ^1^Baycrest Centre for Geriatric Care, Rotman Research Institute, Toronto, ON, Canada; ^2^Department of Medical Biophysics, University of Toronto, Toronto, ON, Canada; ^3^Department of Biomedical Engineering, University of Toronto, Toronto, ON, Canada

**Keywords:** deep learning, fully convoluted neural network, carbon dioxide, respiratory variability, functional MRI, physiological signal analysis, cerebrovascular reactivity (CVR)

## Abstract

**Introduction:**

In the context of functional magnetic resonance imaging (fMRI), carbon dioxide (CO_2_) is a well-known vasodilator that has been widely used to monitor and interrogate vascular physiology. Moreover, spontaneous fluctuations in end-tidal carbon dioxide (PETCO_2_) reflects changes in arterial CO_2_ and has been demonstrated as the largest physiological noise source for denoising the low-frequency range of the resting-state fMRI (rs-fMRI) signal. However, the majority of rs-fMRI studies do not involve CO_2_ recordings, and most often only heart rate and respiration are recorded. While the intrinsic link between these latter metrics and CO_2_ led to suggested possible analytical models, they have not been widely applied.

**Methods:**

In this proof-of-concept study, we propose a deep-learning (DL) approach to reconstruct CO2 and PETCO2 data from respiration waveforms in the resting state.

**Results:**

We demonstrate that the one-to-one mapping between respiration and CO_2_ recordings can be well predicted using fully convolutional networks (FCNs), achieving a Pearson correlation coefficient (r) of 0.946 ± 0.056 with the ground truth CO_2_. Moreover, dynamic PETCO_2_ can be successfully derived from the predicted CO_2_, achieving r of 0.512 ± 0.269 with the ground truth. Importantly, the FCN-based methods outperform previously proposed analytical methods. In addition, we provide guidelines for quality assurance of respiration recordings for the purposes of CO_2_ prediction.

**Discussion:**

Our results demonstrate that dynamic CO_2_ can be obtained from respiration-volume using neural networks, complementing the still few reports in DL of physiological fMRI signals, and paving the way for further research in DL based bio-signal processing.

## 1. Introduction

Carbon dioxide (CO_2_) is a potent vasodilator used that has been shown to rely mainly on the nitric oxide pathway to increase arterial diameter (Pelligrino et al., 1999; Najarian et al., 2000; Peebles et al., 2008; Iadecola, 2017). Blood-vessel diameter is highly sensitive to the surrounding CO_2_ concentration, with increasing CO_2_ partial pressures leading to linear increases in both vessel diameter and flow (Hülsmann and Dubelaar, 1988; Komori et al., 2007). In Komori et al. for example, this increase was shown to be 21.6% for arteriolar diameter and 34.5% flow velocity for a 50% change in CO_2_ partial pressure in rabbit arterioles (Komori et al., 2007). The partial pressure of carbon dioxide (PCO_2_) is the measure of CO_2_ within arterial or venous blood. It often serves as a marker of sufficient alveolar ventilation within the lungs. Under normal physiologic conditions, the value of PCO_2_ ranges between 35 and 45 mmHg, or 4.7–6.0 kPa. Typically the measurement of PCO_2_ is performed *via* arterial blood gas, but the end-tidal pressure of CO_2_ (PETCO_2_) is related to intravascular PCO_2_ through a linear relationship under steady-state conditions (Peebles et al., 2007, 2008), allowing arterial PCO_2_ to be estimated from PETCO_2_.

Dynamic CO_2_ recordings have multiple utilities and implications. In the past decades, the CO_2_-driven functional magnetic resonance imaging (fMRI) response has been the preeminent method for mapping cerebrovascular reactivity (Blockley et al., 2017; Chen, 2018; Chen and Gauthier, 2021). Wise et al. first reported the contribution of spontaneous fluctuations in arterial PCO_2_ to the resting-state fMRI (Wise et al., 2004). Chang et al. followed up this work by demonstrating the potential relationship between PETCO_2_ and respiratory-volume variability (RVT) (Chang and Glover, 2009). Using recordings of spontaneous PETCO_2_ variations, Golestani et al. determined the fMRI response function that links PETCO_2_ to the resting-state blood-oxygenation level dependent (BOLD) signal (Golestani et al., 2015), and also demonstrated PETCO_2_ as the primary source of physiological noise in resting-state BOLD. It has even been used to demonstrate the possible existence of neuronally-motivated vascular networks in the brain (Bright et al., 2020). Furthermore, Chan et al. (2021) found that PCO_2_ (not PETCO_2_) fluctuations also contribute significantly to resting-state BOLD signal variability (Chan et al., 2020). While the mid-breath PCO_2_ does not reflect intravascular PCO_2_, PETCO_2_ does provide a quantitative estimate of arterial PCO_2_, and is more widely used in fMRI experiments for the purposes of denoising (Murphy et al., 2013) and CVR mapping (Pinto et al., 2020). The substantial influence of dynamic PETCO_2_ fluctuations on resting-state (Golestani and Chen, 2020) and dynamic functional connectivity has been demonstrated recently (Nikolaou et al., 2016). Dynamic CO_2_ can also allow vascular lag structures to be estimated, providing an important metric for assessing vascular health (Champagne et al., 2019). Given the unique variance explained by PCO_2_ and PETCO_2_, it is safe to say that dynamic CO_2_ is a useful thus desirable metric for those working with resting-state fMRI data.

Despite the increasing realization of the value of CO_2_ recordings, it is often impossible to obtain recordings of CO_2_ during an fMRI session. Most study sites are not equipped with an MRI-compatible capnometer that also facilitates continuous recording of PCO_2_. Moreover, the many thousands of legacy fMRI data sets (e.g., Human Connectome Project, UK Biobank) certainly do not include CO_2_ recordings. On the other hand, respiratory volume variations, which had previously been related to PETCO_2_ variations, are more readily available thanks to the incorporation of respiratory-volume belts in modern MRI systems. RVT was first introduced by Birn et al. as a noise source in fMRI that introduces unique signal variability (Birn et al., 2006). Today, while RVT measurements during fMRI sessions are increasingly common, they are still unavailable in large-scale studies and legacy data sets. As a possible solution, recent work by Salas et al. (2020) demonstrated that the RVT time series can in principle be reconstructed from fMRI data using a convolutional neural network (CNN).

Chang et al. previously showed that PETCO_2_ can be related to RVT through a respiratory-response function (Birn et al., 2008). However, this relationship has been difficult to reproduce in resting-state conditions, as we will show with our data. In the resting state, not only is it impossible to derive quantitative CO_2_ values from respiratory volume, it is also difficult to obtain a deterministic relationship between dynamic patterns of respiratory volume and CO_2_ variation. Thus, in this study, we also use the principle of DL, but our focus is to bridge the gap between respiratory and CO_2_ recordings. Our aim is to demonstrate the feasibility of using DL to produce dynamic CO_2_ waveforms from the respiratory time series.

### 1.1. Background on neural networks

In the majority of DL methods for neuroimaging, 2D inputs are used to produce 2D outputs (Zhu et al., 2019). Image-to-image translation is used for cross-modality conversion, denoising, super-resolution and reconstruction (Kaji and Kida, 2019). Our problem entails the estimation of a 1D signal from another 1D signal, and within this context, past research has used convolutional neural networks (CNNs) and recurrent neural networks (RNNs). Traditional CNNs consist of convolutional layers followed by fully connected layers (dense layers) terminating the network (Rawat and Wang, 2017). As CNNs are the most successful type of DL model for 2D image analysis, and physiological signals are 1D time-series data, some have converted 1D signals to 2D data to be fed into a CNN, and have obtained good results (Shah et al., 2022). The advantage of using 1D CNNs over 2D CNNs and RNNs is the significant reduction in the number of training parameters, which is helpful when the training data is limited (as the application at hand). Applications of 1D CNNs include ECG classification and anomaly detection in biomedical signals (Kiranyaz et al., 2021). Salas et al. pioneered the use of 1D CNN for estimating physiological fluctuations in fMRI, an application closely related to ours. They segmented the BOLD fMRI signals into fixed time-windows and fed them into a CNN, where the dense layer predicted a single point of the respiration waveform at the center of the window. To predict the entire time series, all the time-windows have to be separately propagated through the network, entailing high complexity and computational cost. Moreover, commonly found respiration-belt recordings have variable lengths, which are incompatible with the use of dense layers.

In this work, we implemented a type of CNN known as fully convolutional networks (FCNs) (Long et al., 2015). A FCN is simply a traditional CNN without any fully connected layers. Fully convolutional layers in FCN permit the use of variable-length input and also minimizes the computational cost. Previously, a 1D U-net (a type of FCN that includes skip connections) was implemented for reconstructing low-frequency respiratory-volume signals from fMRI time-series data (Bayrak et al., 2020). Here, we demonstrate the use of simple FCNs (without skip connections) for predicting 1D data wherein the encoder-decoder architecture exploits the latent space to streamline the prediction of CO_2_ traces from respiration-belt signal, in the presence of limited training data.

## 2. Methods

### 2.1. Data acquisition

We recorded percent-CO_2_ (%CO2) fluctuations and respiratory bellows simultaneously in a group of 18 healthy adults (age 20–38 years) using the Biopac System (Biopac Inc., Goleta, CA, USA). The Biopac respiration belt was positioned below the ribcage, and detects respiratory depth by sensing abdominal circumference changes. %CO_2_ data were acquired through gas lines attached to masks affixed to subjects' faces. The Biopac %CO_2_ module (CO2100C) is calibrated to measure %CO_2_ concentration in the range of 0 to 10%. In total, the available data set consisted of 136 resting-state recordings from different subjects, which were 10.8 min long on average (min = 7.2 min, max = 16.1 min). The procedure was approved by the Research Ethics Board of Baycrest (REB# 11–47, approved Dec. 2011–19). To the best of our knowledge, this is the largest data set of its kind in existence.

### 2.2. Data preprocessing

The preprocessing steps consist of (1) low-pass filtering both respiration and CO_2_ waveforms (f < 1 Hz) and (2) correcting the delay between %CO_2_ and respiration signal by cross-correlation. The low pass filter's cutoff frequency was determined based on the respiratory rate of an individual (0.2–0.4 Hz). The delay between %CO_2_ and respiration waveforms were corrected by shifting the %CO_2_ time course by the time lag yielding the maximum negative cross-correlations between it and the respiration waveform. We found that across all cases, to achieve this, the %CO2 time course had to be shifted to the left (backwards in time) by an average of 8.5 s (with a standard deviation of 1.5 s).

After the delay correction process, we rejected data that yielded absolute Pearson correlations of < 0.4. Recordings were also rejected if their length was < 3 min, too short to allow adequate training. More details on the correlation and data-length threshold are given in the quality assurance section. The respiration belt data was in arbitrary units; hence it was normalized by subtracting the temporal mean and dividing the result by standard deviation. The same procedure was applied to the %CO_2_ waveforms. Further details about the normalization are provided in the next subsection. Both the waveforms were then resampled to 10 Hz and exported in CSV format to be later imported during the training phase of the neural network.

To obtain PETCO_2_ from the normalized %CO_2_ recordings, the peak-detection step [available through SciPy: (Virtanen et al., 2020)] ensures the minimum distance between the two peaks is twice the sampling interval. In other words, we assumed the time between two exhales is at least 2 s, which is consistent with our recorded respiratory intervals (3–5 s per breath). Moreover, the lower limit of the amplitude of the peak was set to be 0.3, and negative peaks are also rejected.

#### 2.2.1. Data normalization

As previously mentioned, both %CO_2_ and respiration-belt data were demeaned and normalized to unit standard deviation (such that SD = 1). The respiration data is fluctuations in voltage transduced from expansions and contractions of the belt. As such, it varies with slight variations in belt tightness and positioning, and needs to be normalized across subjects to achieve inter-subject consistency. In part due to the need of using normalized respiration as the independent variable, this latter would encode no quantitative %CO_2_ information. That is, there could be a many-to-one relationship between normalized respiration and unnormalized CO_2_. To mitigate this issue, we demeaned and normalized the %CO_2_ time series in the same manner. In this manuscript, all the further mentions of CO_2_ denote normalized %CO_2_, unless stated otherwise.

#### 2.2.2. Quality assurance

A critical part of successful application of machine learning is quality assurance (QA) of the training and testing data. It is more probable to find noise in respiration data, wherein artifacts such as subject movement and talking can easily confound respiration-belt recordings. Moreover, if the participant does not consistently breathe from the abdomen, the respiration belt data may not correspond well with the CO_2_ data. During the data-collection phase, useful precautions include ensuring that the respiration belt and CO_2_ gas lines are properly connected. Such precautions not only reduce the unwanted waveforms but also increase the feasibility of machine-learning approaches. To discard the undesirable recordings, we have evaluated our data based on the criteria below. Nonetheless, it is informative to use data containing some level of noise and artifact for the purposes of representativeness. Therefore, the threshold used in the rejection process is generously selected.

##### 2.2.2.1. Length of the recording

**Figure 1:**
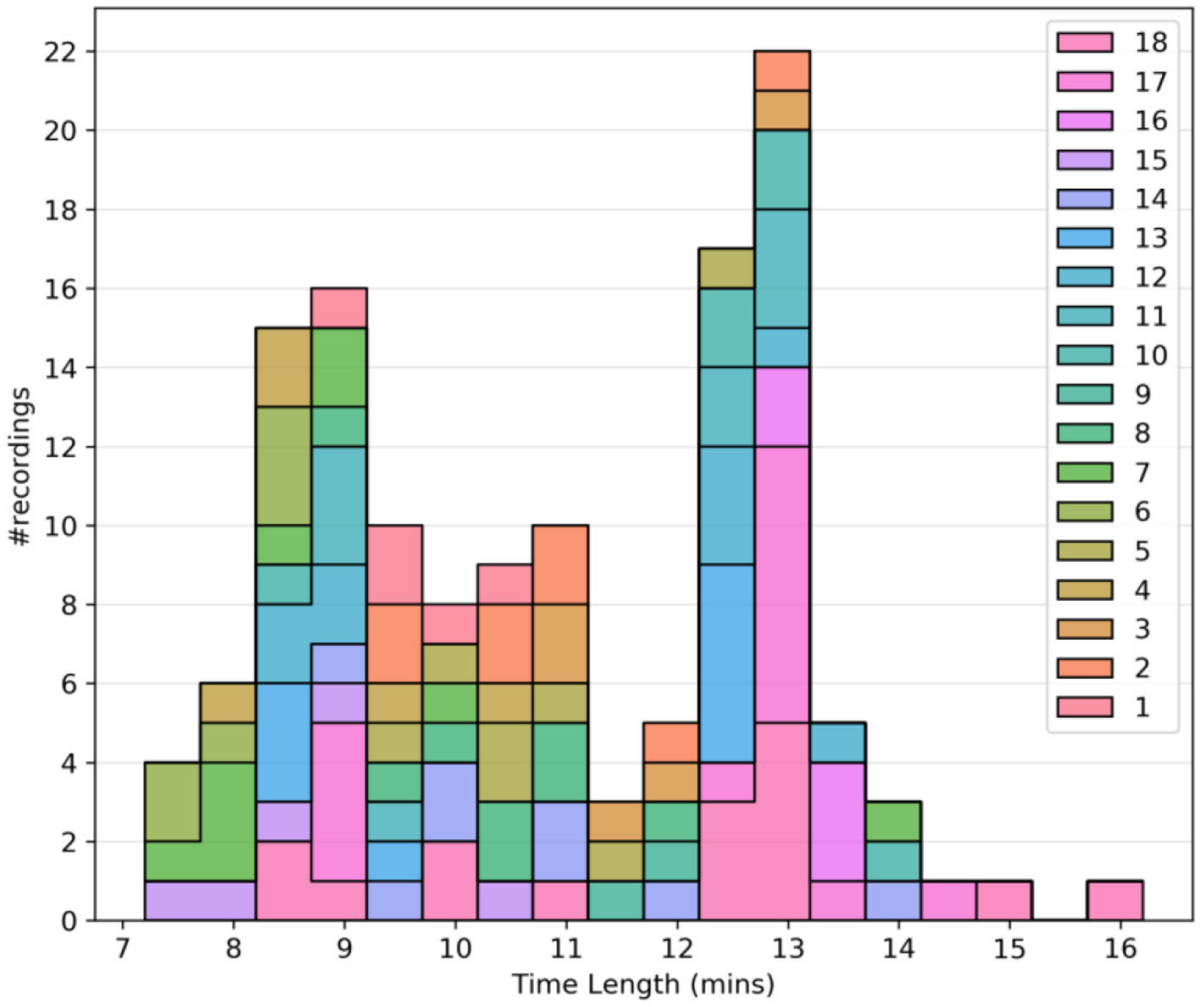
Quality assurance metrics: Histogram plot of the time length of recordings after time length thresholding. Different colors are used to separate the subjects.

In general, for our approach, longer data sets are more desirable. It was observed that all the recordings were either < 3 min or more than 6 min in length, drawing a clear distinction between test recordings and usable recordings. Thus, the lower limit for the time length was set to 3 min. Figure 1 shows the histogram plot of all the recordings after the time-length thresholding.

##### 2.2.2.2. Pearson correlation coefficient

**Figure 2:**
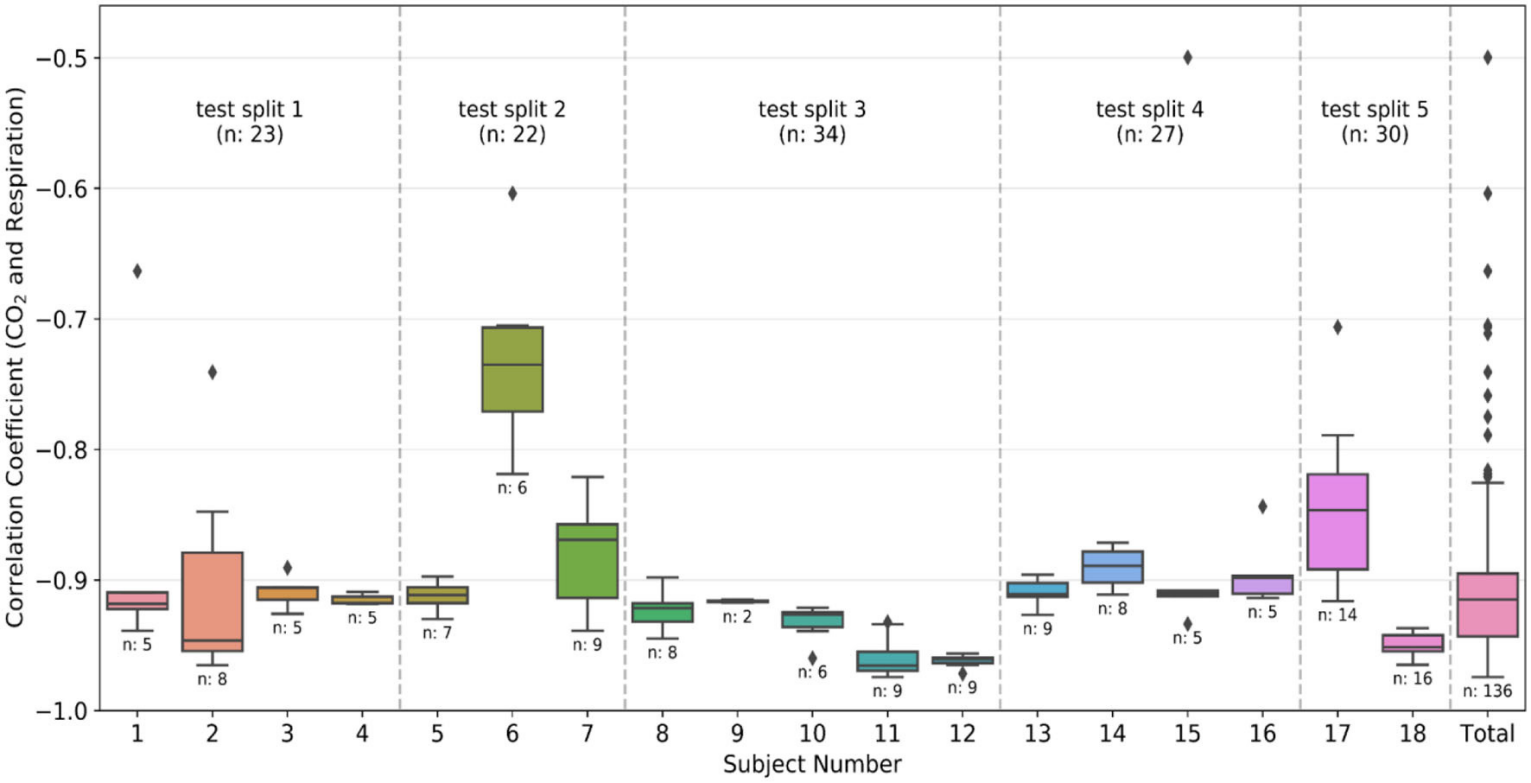
Quality assurance metrics: Box plots of the correlation coefficient between CO_2_ and respiration waveforms from each individual subject and the total data after preprocessing. The number of recordings available for each subject is also given below the box plot. The divisions created by the dashed line show the groups made during the k-fold split of the dataset. The group number is the same as the test split number, and the total number of recordings in the group is also provided in the plot. The color-coding is the same as Figure 1.

As previously mentioned, Pearson's correlation (r) between the respiration belt and CO_2_ time courses is used for initial QA purposes. The threshold for the absolute value of correlation between CO_2_ and respiration is −0.4, as respiratory volume and CO_2_ are expected to be negatively associated. This limit was empirically determined through manual review of the recordings. Figure 2 shows that even though the threshold was −0.4, there were no recordings with r between −0.4 and −0.5, only one recording with r = −0.5 and most of the recordings had an r value of < -0.6.

##### 2.2.2.3. Low-frequency noise in the waveforms

Within the 0.1–0.5 Hz frequency band, noise in the respiratory and CO_2_ waveforms can impair our ability to relate the two waveforms, even if the recording-duration and correlation-coefficient thresholds are met. Such noise most likely originates from faulty attachment of the respiration belt and from drifts in the recording modules. As it could potentially overlap with breathing frequency, it cannot be separated from the signal by using filters. However, this type of noise can be identified through a mismatch in the low-frequency portion (< 0. 2Hz) of the power spectra of CO_2_ and respiration, as shown in Supplementary Figure 1. This type of noise is also reflected in the signal time series as periodic decreases or increases in the amplitude of signal. Conversely, an exemplary data set is shown in Supplementary Figure 2.

#### 2.2.3. Neural network

Obtaining the CO_2_ concentration from the respiration waveform is a 1D-to-1D (time series to time series) translation problem, which is modeled using a 1D fully convolutional encoder-decoder architecture. This modeling is analogous to prevalent image-to-image translation or semantic segmentation using 2D FCNs (Long et al., 2015; Alotaibi, 2020). However, most recent works in image-to-image translation problems involve adversarial training (Pang et al., 2022), which is notoriously hard especially with limited data. Thus, adversarial training is excluded in this paper. Constructing a deep neural network often involves trial and error for tuning hidden layers. To find an optimum number of hidden layers in the network, several FCNs architectures are investigated, until overfitting was observed (test phase error increases with increasing network complexity). All codes are written in Python and use the PyTorch library, and would be publicly available on GitHub.

##### 2.2.3.1. FCN architecture

Input to the network was an array of size C x L, where the number of input channels, C = 1 and L is the length of recording. Although the respiration recordings were normalized using standard deviation, the resultant data range still varied between data sets. To bound the respiration amplitude within a fixed range, the respiration array was further normalized using the tanh operator before being passed on to the fully convolutional layers. We implemented four different FCN architectures, each having one (FCN-1L), two (FCN-2L), four (FCN-4L) and six (FCN-6L) convolution layers, respectively, between the input and output layers.

**Figure 3:**
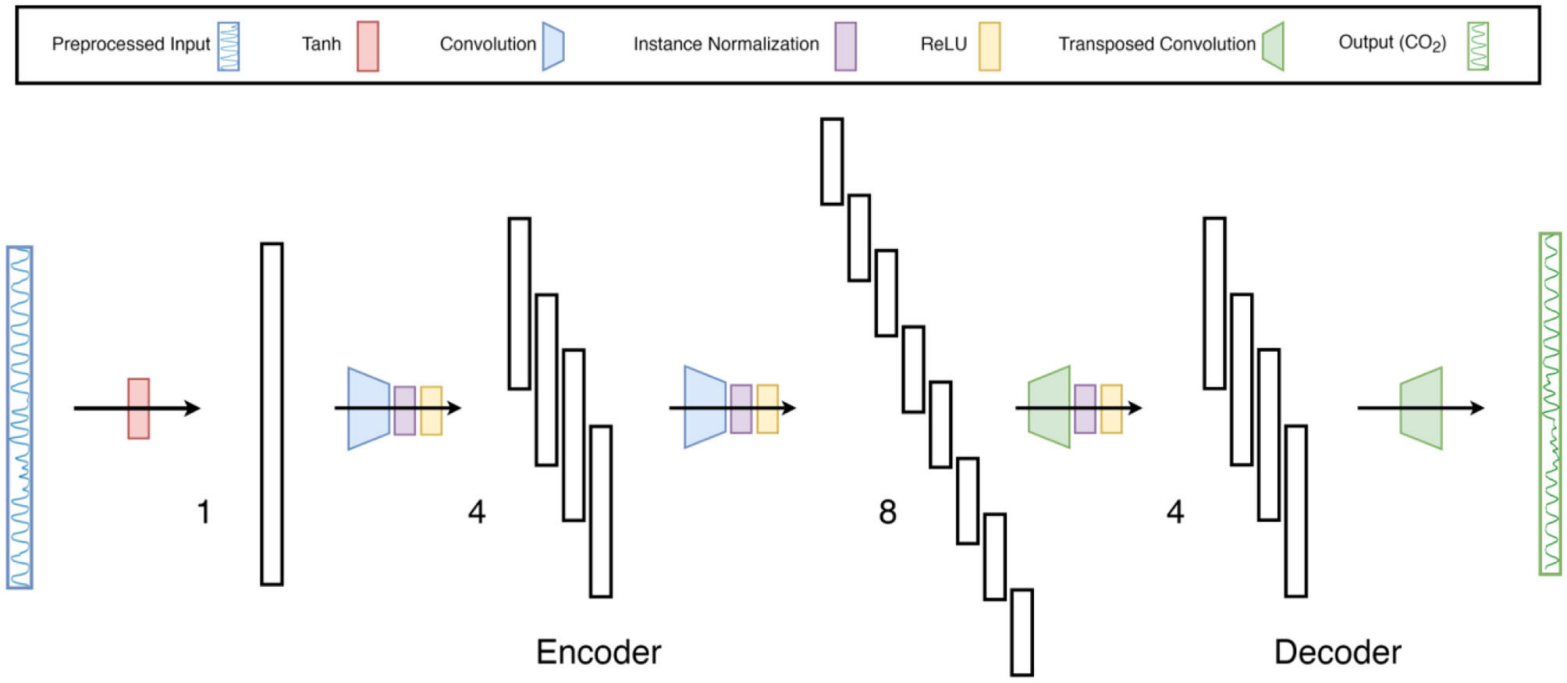
Neural-network architecture: 4-Layer Fully Convolutional Network. The architecture shown here is a type of encoder-decoder neural network consisting of fully convolutional layers, followed by instance normalization and ReLu non-linearity. The last layer does not contain normalization and activation function as it is a regression problem. Moreover, the input is first normalized using tanh activation function to constrain the input data between −1 and 1. The numbers 1, 4 and 8 indicate the number of filters per layer.

FCN-1L consists of a single convolution operation with a kernel of length 7 and replicate padding of 3 on both sides (head and tail) of the input waveform. The kernel length is chosen to balance model complexity with prediction accuracy. FCN-2L encodes the tanh-normalized respiration waveform by convolving it with a 4 × 7 kernel (4 kernels of length 7) with a stride of 2, which means the input is downsampled by a factor of 2. This is followed by ReLU nonlinearity (activation function) and finally a transposed convolution to decode the hidden layer into CO_2_. Both the convolution and transposed convolution are performed with a stride of 2, which replaces the need for a pooling layer to downsample the output of convolutional layers and an unpooling layer to upsample the output of transposed convolutional layers. Similarly, FCN-4L consists of 2 convolution and 2 transposed convolutional layers, and FCN-6L architecture adds another 1 layer to both encoder and decoder sections. The network architecture of FCN-4L is shown in Figure 3.

##### 2.2.3.2. Loss function

We also experimented with two different loss functions. The first loss function is the mean squared error (MSE) computed between the measured and predicted CO_2_ waveforms, which is widely used in regression problems (Equation 1). However, as the regression was performed between the waveforms of pseudo-periodic nature, it was observed that the network learned to predict zero-crossings extremely well, but the extremities were left underfitted, lowering the scores of PETCO_2_ predictions. To rectify this problem, a second loss function, the weighted MSE (MSE_Wgt_), was introduced Equation 2), with the weights set to the normalized amplitudes of the ground truth CO_2_ waveform for each timepoint. The weighting provides higher preference to the peaks, and hence we hypothesized that it would provide better results for PETCO_2_.


(1) $$\begin{array}{l} MSE=\frac{1}{L}\sum_{i=1}^{L}{\left(y_{i}-{ŷ}_{i}\right)}^{2} \end{array}$$

(2) $$\begin{array}{l} MSE_{Wgt}=\frac{1}{L}\sum_{i=1}^{L}{\left[\left(y_{i}-{ŷ}_{i}\right)/|y_{i}|\right]}^{2} \end{array}$$

where, y_i_ and y_i_ are the predicted and ground truth CO_2_ respectively for the *i*^th^ time point, and L is the length of the recording. Networks trained with the weighted cost function are denoted by the postfix “-Wgt.”

#### 2.2.4. Training

The 18 subjects were split into 5 subsets (splits), and the training was executed using the k-fold cross-validation strategy. It is typical to use either 10-fold or 5-fold cross-validation as it generally results in a model with low bias, modest variance and low-computational cost compared to leave-one-out cross-validation strategy (Rodriguez et al., 2010). In our dataset, as the number of subjects is relatively limited, we opted for k = 5, and each time one subset was left out from the training phase to be used in testing the accuracy of the network. Each subject can have multiple recordings, and the data was divided based on the subjects (and not recordings) to ensure that the training and testing data has no scans sharing a common subject. The divisions created by dotted lines in Figure 2 correspond to the different splits. As visible in the figure, the splits contain data from 2, 5, 4, 4, and 3 subjects, yielding total numbers of 30, 34, 27, 23, and 22 recordings, respectively. Each split has a different number of total recordings, which enhances the generalizability of the results. We implemented two training strategies.

##### 2.2.4.1. Method 1. Equal-length data segments

In this method, we formatted the training data as an array of equal-sized data segments obtained by segmenting the input recordings. As the training was performed on a GPU, the computation parallelized in the tensor with multiple batches, reducing the training time. We used the chunk size of 90 s and a batch size of 256. The drawback of this method is the unavoidable error introduced due to edge effects during convolution, which is proportional to the number of chunks.

##### 2.2.4.2. Method 2. Variable-length data segments

In this method the input array length could be of variable sizes. The drawback of using variable-length input is that it prevents us from grouping the data in batches for parallel processing in the GPU. On the positive note, unlike in Method 1, Method 2 precludes the segmenting-induced edge effects. We implemented both methods. The training time was < 20 s irrespective of the network type or training method. All the networks were trained using Adam optimizer for 15 epochs. Hyperparameters corresponding to the optimizer like learning rate and decay rate were fine-tuned manually for each network. In total, we trained four FCNs, each using two loss functions, on the 5-fold split data. The training was performed on a 12GB GeForce GTX TITAN X GPU. All networks used < 500MB GPU memory during the training phase.

##### 2.2.4.3. Reference methods

To the best of our knowledge, there have been no previous attempts to derive the CO_2_ waveform from respiratory traces using machine learning. To establish the performance of our approach against a possible alternative, we employed two reference methods. First, based on previous work by Chang and Glover (2009), defining a PETCO_2_ as the convolution of RVT with RRF (and then normalized, negated and shifted temporally for maximum cross-correlation). This is referred to as the RVTRRF method, described by Equation 3. RVT was estimated from respiration waveform as detailed in Birn et al. (2008).


(3) $$PETCO_{2}{}^{\prime }\left(t\right)=RVTRRF\left(t\right)=RVT{\left(t\right)}^{*}RRT(t)$$

where $PETCO_{2}{}^{\prime }\left(t\right)$ is the estimated PETCO_2_. RRF is the respiratory response function, and ^*^ denotes convolution. Similar to what was done previously (Chang and Glover, 2009), at the testing stage, we corrected the lag between RVTRRF [${\text{PETCO}}_{2}^{{}^{\text{′}}}$(t)] and PETCO_2_ using the maximum cross-correlation between the two signals, where the time shift was allowed to vary between −120 and 120 s. Moreover, to maintain the scaling of PETCO_2_ as obtained from neural networks, we normalized and demeaned RVTRRF with the standard deviation and mean of PETCO_2_.

Second, defining a linear-regression (LR) model relating CO_2_ to respiratory volume (Equation 4), and PETCO_2_'(t) is extracted from the CO_2_ time courses (measured using the Biopac system in this case).


(4) $$CO_{2}{}^{\prime }\left(t\right)=\beta \;\cdot \;Resp\left(t\right)+\varepsilon$$

where CO_2_' is the estimated CO_2_, Resp(t) is the respiratory-belt signal, ε is the intercept, and β is the linear weighting factor derived from the “training data,” and the LR model could be understood as a single convolutional operation with a unit kernel size, making it similar to a machine learning linear regression problem. The training and testing partitioning are as described for the FCNs. MSE loss function was backpropagated similar to the FCNs.

##### 2.2.4.4. Evaluation criteria

For the evaluation, the Pearson correlation coefficient (r), mean squared error (MSE), mean absolute error (MAE) (Equation 5) and mean absolute percent error (MAPE) (Equation 6) were calculated between (1) predicted CO_2_ and ground-truth CO_2_, (2) predicted PETCO_2_ and ground-truth PETCO_2_. As the MAPE is sensitive to zero crossings, it was only calculated between the predicted PETCO_2_ and ground-truth PETCO_2_.


(5) $$\begin{array}{l} MAE=\frac{1}{L}\sum_{i=1}^{L}\left(\left|y_{i}-{ŷ}_{i}\right|\right) \end{array}$$

(6) $$\begin{array}{l} MAPE=\frac{1}{L}\sum_{i=1}^{L}\left(\left|\frac{y_{i}-{ŷ}_{i}}{y_{i}}\right|\right) \end{array}$$

We also performed statistical comparisons amongst correlation coefficients and MSE values obtained using all FCN and reference methods using the Kruskal-Wallis test, corrected for false-discovery rate.

The final validation is inspired by a practical application of CO_2_ recordings, namely examining the relationship between PETCO_2_ and resting-state fMRI time series. For this we include 3 cases acquired from each of the 2 healthy young subjects (male, age = 25 and 33 years). All data were acquired using a Siemens TIM Trio 3 T system and a 32-channel head coil. CO_2_ was acquired during these scans as described earlier. That is, each dataset contains the following:

- Case 1: spin-echo EPI, TR = 323 ms, TE = 45 ms, flip angle = 90°, 2,082 frames, voxel size = X: 3.48 mm, Y: 3.48 mm, Z: 6.25 mm;
- Case 2: gradient-echo EPI, TR = 323 ms, TE = 30 ms, 2,230 frames, voxel size = X: 3.48 mm, Y: 3.48 mm, Z: 6.25 mm;
- Case 3: simultaneous multi-slice gradient-echo EPI, TR = 323 ms, TE = 30 ms, flip angle = 40°, 2,230 frames, voxel size = X: 3.48 mm, Y: 3.48 mm, Z: 6 mm;

Preprocessing steps include: (1) filtering to 0.01–0.1 Hz band with AFNI (Cox, 1996); (2) spatial smoothing with a 5 mm kernel (Jenkinson et al., 2012) (3) Discard the first 5 volumes in each scan to allow the brain to reach a steady state. All recorded and FCN-generated CO_2_ and PETCO_2_ time courses were low-pass filtered to 0.01–0.1 Hz to match the temporal resolution of the respective fMRI data.

## 3. Results

**Figure 4:**
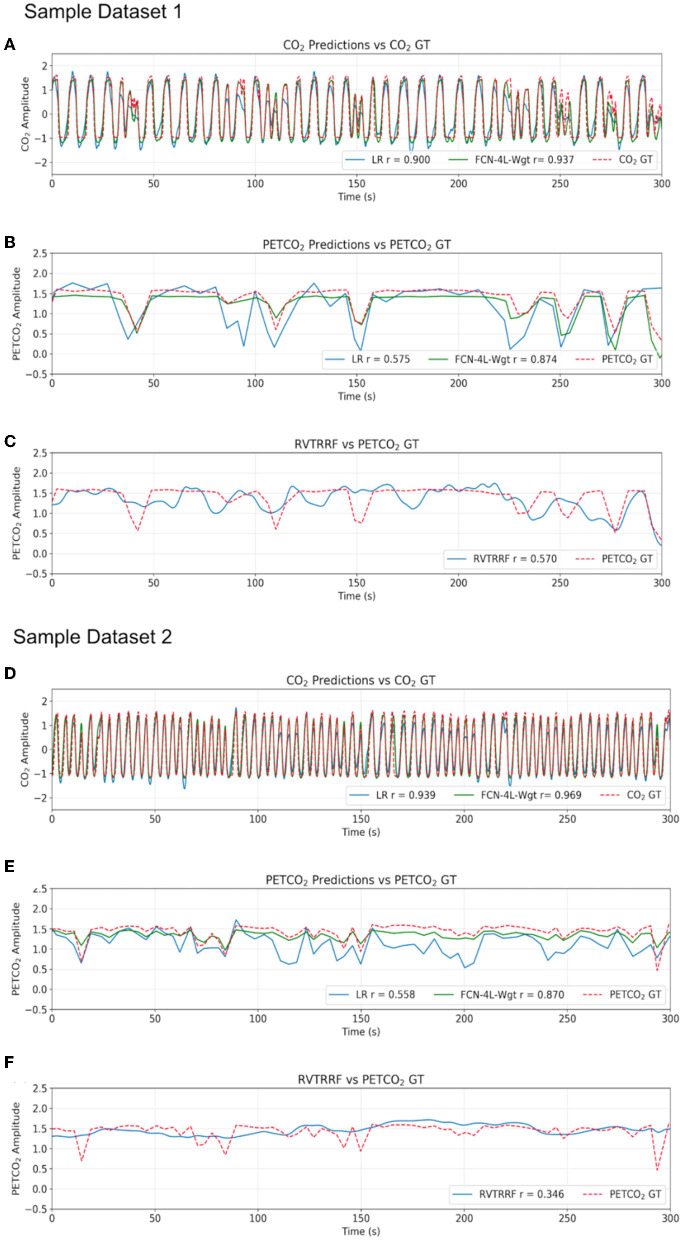
Qualitative comparison of resultant outputs. Two different sample predictions are shown from the test dataset, and for each of the example, comparisons are made between **(A, D)** the CO_2_ prediction and ground truth (GT), **(B, E)** the PETCO_2_ prediction from the reference linear regression model (LR), FCN-4L-Wgt model and the GT, and **(C, F)** PETCO_2_ estimated from RVTRRF and the PETCO_2_ GT.

Results for two representative data sets are shown in Figure 4. Method 1 (equal data length) adds no extra benefit to the training process and results in poor performance due to possible truncation effects in training data. Thus, all the results provided here correspond to Method 2. The results are shown in Figure 4 and summarized in Table 1. The best method, as determined by the lowest error terms (MSE, MAE, MAPE) and highest Pearson correlation (r) is indicated in bold. The predicted and ground-truth PETCO_2_ show excellent visual agreement for FCN-4L-Wgt (Figure 4B). From Table 1, we can see that the CO_2_ estimation error obtained from FCN-4L and FCN-4L-Wgt architecture are identical, with the errors corresponding to PETCO_2_ being slightly lower in the latter case. Since r is unaffected by scaling and translation, and since the LR model involves only scaling and translation, the modeling step would not improve r. Strangely, the RVTRRF model performs worse than the LR model (for PETCO_2_), suggesting that estimating PETCO_2_ from the peaks of the CO_2_ (and hence respiration) waveform may be more robust.

**Table 1:** Quantitative assessment of various approaches and network structures.

**Average across all 5 splits**	**RVTRRF**	**LR**	**FCN-1L**	**FCN-2L**	**FCN-4L**	**FCN-6L**	**FCN-4L-Wgt**
r CO_2_	-	0.901 ± 0.061	0.931 ± 0.055	0.922 ± 0.06	0.946 ± 0.054	0.944 ± 0.055	0.946 ± 0.056
r PETCO_2_	0.256 ± 0.132	0.311 ± 0.239	0.443 ± 0.261	0.45 ± 0.262	0.5 ± 0.266	0.461 ± 0.235	0.512 ± 0.269
MSE CO_2_	-	0.19 ± 0.103	0.138 ± 0.094	0.151 ± 0.101	0.108 ± 0.097	0.11 ± 0.097	0.106 ± 0.101
MSE PETCO_2_	0.032 ± 0.028	0.026 ± 0.021	0.02 ± 0.018	0.019 ± 0.017	0.018 ± 0.017	0.02 ± 0.018	0.017 ± 0.017
MAE CO_2_	-	0.337 ± 0.079	0.269 ± 0.077	0.276 ± 0.08	0.223 ± 0.076	0.227 ± 0.081	0.213 ± 0.08
MAE PETCO_2_	0.121 ± 0.055	0.112 ± 0.045	0.094 ± 0.04	0.093 ± 0.039	0.081 ± 0.035	0.085 ± 0.038	0.08 ± 0.036
MAPE PETCO_2_	0.125 ± 0.109	0.112 ± 0.084	0.094 ± 0.077	0.095 ± 0.078	0.085 ± 0.073	0.089 ± 0.074	0.084 ± 0.077

RVTRRF, RVT convolved with RRF; LR, linear regression; FCN-XL, “X” layered FCN used; -Wgt, with weighted MSE cost function. The parameters used in the assessment include: the correlation coefficient (r), the mean-squared error (MSE), the mean absolute error (MAE) and the mean-absolute percent error (MAPE). Each metric was calculated for every recording in the test set across all five splits. The mean and standard deviation (mean ± std) were calculated for all the metrics in each test split. Likewise, the average of (mean ± std) was taken across all the 5 splits and displayed in this table.

**Figure 5:**
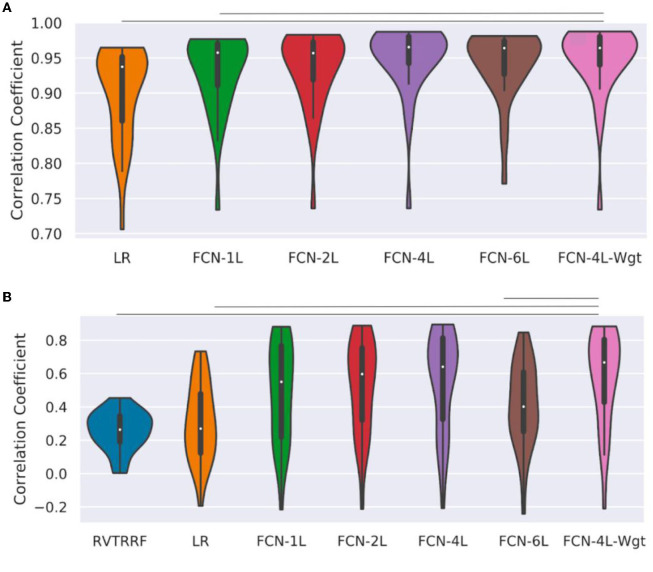
Performance of different methods: Distribution of correlation coefficients (r) on test dataset, where r is computed between **(A)** ground-truth and predicted CO_2_, and **(B)** the ground-truth and predicted PETCO_2_ obtained on the test dataset (for one of the five splits) is compared for different models used in the study and shown in the form of a bean plot. The median r for each method is shown as a white dot at the centers of the distributions. The horizontal lines indicate statistically significant differences between the two approaches at the ends of the lines. The FCN-4L-Wgt approach is significantly superior than the RVTRRF and LRF approaches for predicting CO_2_, and better than FCN-6L additionally in predicting PETCO_2_, shown by the significantly higher r values.

Figure 5 shows the r distribution across the entire test dataset for one of the five splits. The LR method is outperformed by all FCN methods (and significantly so by FCN-4L-Wgt) for CO_2_ prediction. The difference between FCN-4L and FCN-4L-Wgt is not noticeable in the case of CO_2_ prediction, but overall, FCN-4L-Wgt achieved the highest r values, while FCN-6L achieved the lowest r variability. However, for PETCO_2_, FCN-4L-Wgt reached higher r values than did FCN-4L, demonstrating the superiority of a weighted loss function. FCN-6L performs worse than all the other FCN networks for PETCO_2_ prediction. However, these differences are not statistically significant, as can also be seen in Table 2, in which every approach is compared to the apparent leader (FCN-4L-Wgt). Note that the RVTRRF method only reached a maximum r score of just below 0.5, substantially lower compared to all FCN networks. As previously mentioned, the r scores for RVTRRF correspond to maximum cross correlation with PETCO_2_, thus the scores are always positive. There is no such limitation for the FCNs, resulting in some network correlation coefficients in the distribution.

**Table 2:** Statistical comparison of various approaches and network structures with FCN-4L-Wgt.

**Metric**	**RVTRRF**	**LR**	**FCN-1L**	**FCN-2L**	**FCN-4L**	**FCN-6L**
r CO_2_	**0.0001**	0.0287	0.0895	−0.9146	0.2189	0.0001
r PETCO_2_	**< 0.0001**	0.0001	0.1646	0.3593	0.8941	0.0071
MSE CO_2_	**< 0.0001**	0.0001	0.1646	0.3593	0.8941	0.0071
MSE PETCO_2_	0.6048	< 0.0001	**0.0001**	**0.0005**	0.1833	**0.001**

Listed at the p values indicate the significance of differences. All tests for PETCO_2_ prediction were performed with 209 degrees of freedom (DOF), and with a DOF of 179 for CO_2_ prediction. All p-values were corrected for multiple comparisons. Entries meeting statistical significance are indicated in bold face.

**Figure 6:**
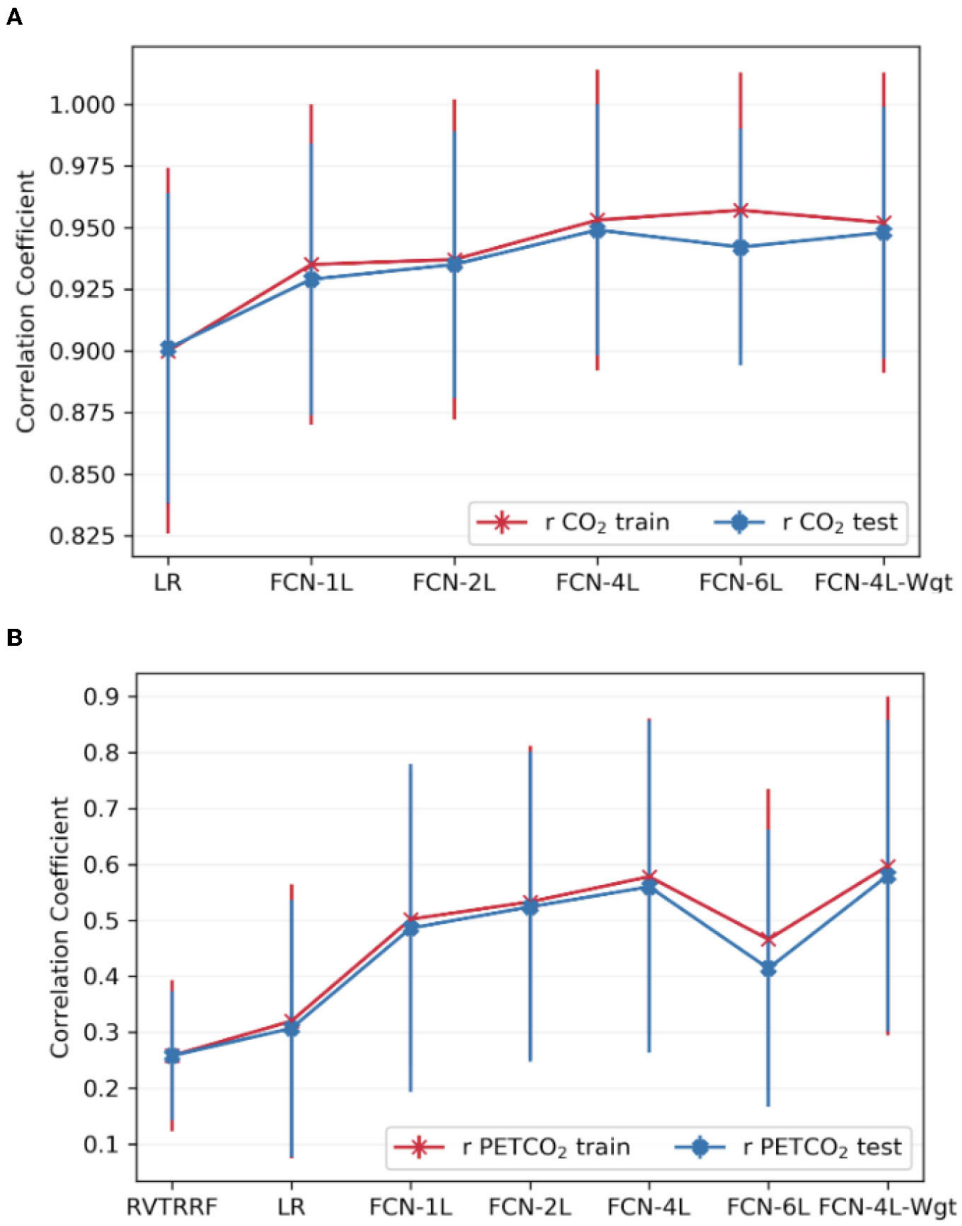
Comparison of model performance on train vs. test datasets. The average Pearson correlation coefficient obtained across one of the splits for **(A)** CO_2_ and **(B)** PETCO_2_ between test and train dataset is shown in the top row. The error bars indicate the standard deviation.

Figure 6 compares the correlation scores between training and testing phase for all the networks. From these plots, it can be inferred that FCN-6L likely overfits the training data, as reflected by a worse performance than that of the other networks (as reflected by a lower r). Since FCN-4L performs better than FCL-2L and doesn't show huge differences between training and testing results, we can say four convolutional blocks are the optimum number for our given training data. Moreover, in our best model, MAPE score for PETCO_2_ is 0.142 (< 0.2), reflective of good prediction performance.

**Figure 7:**
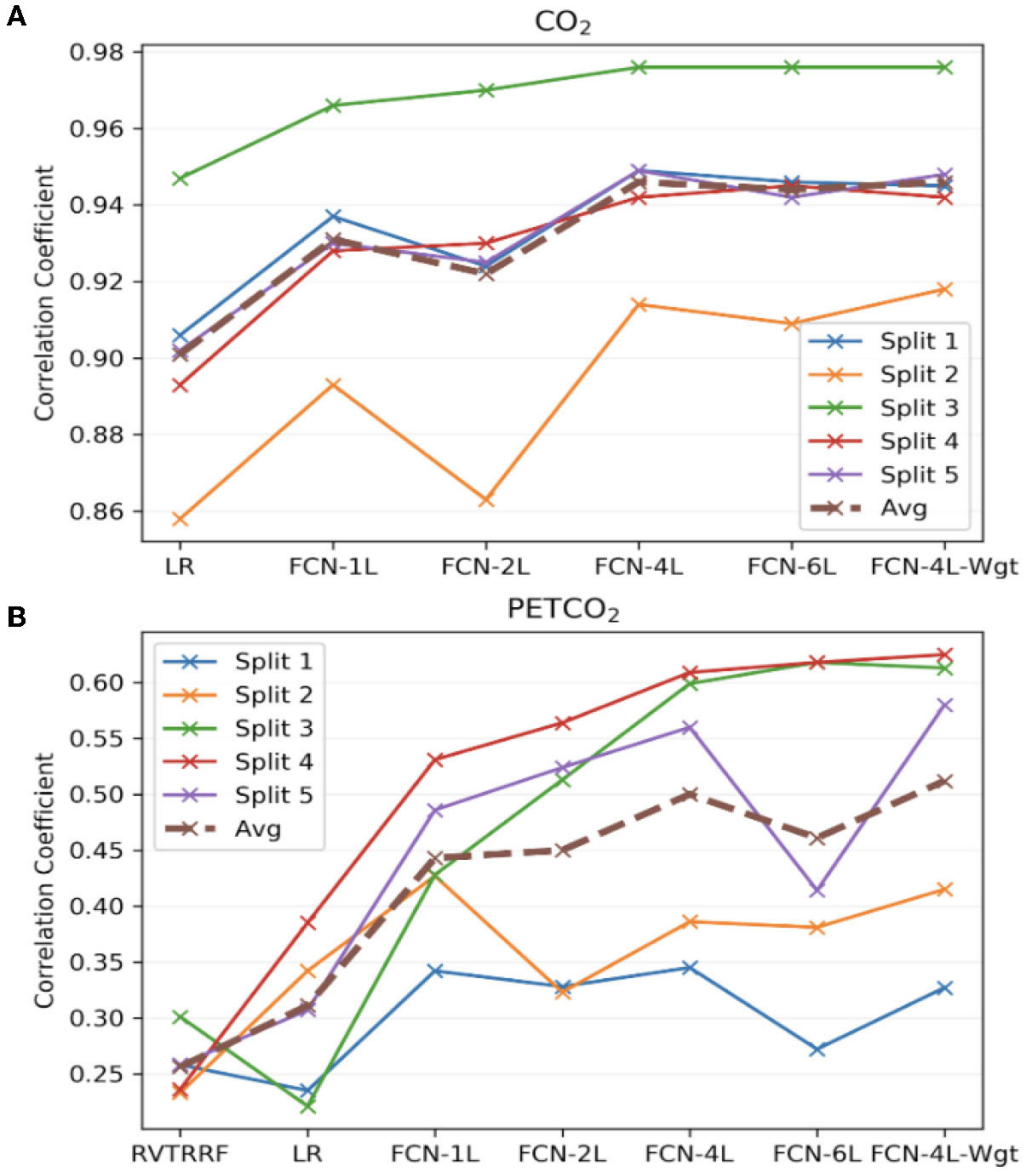
Model performance across the five splits. The correlation coefficients (r) obtained across the five splits and their average for all the models, for **(A)** CO_2_ and **(B)** PETCO_2_ prediction. The split number is the same as the splits shown in Figure 2.

Figure 7 compares the correlation coefficients across the five splits for all the networks. The r-score ranking in the case of CO_2_ prediction does not match with that of PETCO_2_ prediction. In the case of CO_2_, the r for FCN-4L-Wgt closely resemble those of FCN-4L, but the former performed better for PETCO_2_ (in all but one split). Though the best model varied depending on the split number and varies between CO_2_ and PETCO_2_ prediction, FCN-4L-Wgt consistently outperformed other models, exemplified in part by the highest correlation coefficients. The inter-split variability in r is the lowest for the reference methods (RVTRRF and LR) and highest for FCN methods, the various FCN methods themselves do not appear to exhibit different degrees of inter-split performance variability. Moreover, the performance rankings of the various methods are consistent across the splits and in line with the trends observed in Figure 5. Combining the results of Figure 7 with the information in Figure 2, it can be seen that the poor CO_2_-prediction performance for all methods across the second split is due to one subject (subject 6). CO_2_ prediction in Split 3 was best overall. Yet, the LR model performs worst in predicting PETCO_2_ in the 3rd split, reflecting that higher correlation between CO_2_ and respiration does not necessarily translate into higher correlation between PETCO_2_ and respiration. This point is further demonstrated by contrasting r scores of PETCO_2_ and CO_2_ for the LR approach in the remaining splits.

**Figure 8:**
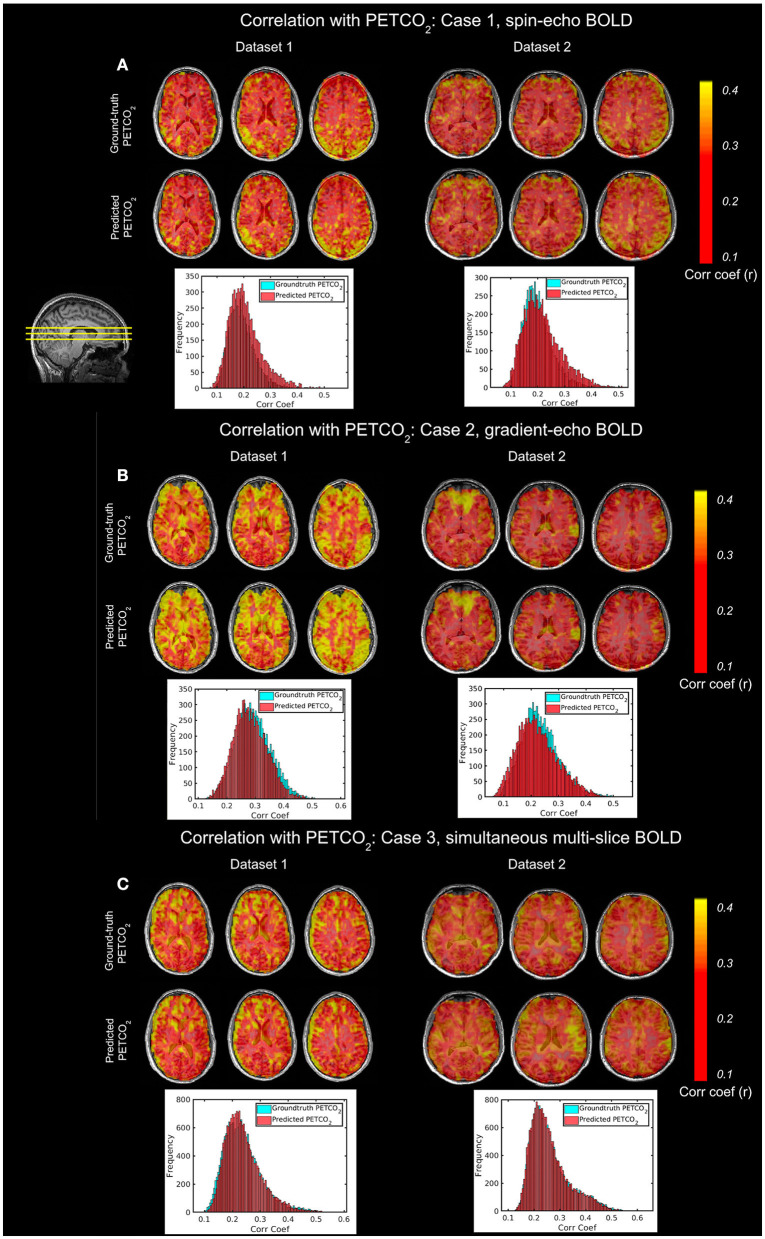
Comparison of ground-truth and predicted PETCO_2_ correlations. Data from 2 different subjects, imaged over multiple sessions [**(A–C)**, respectively] are shown. In each case, the peak cross-correlation maps generated using the ground-truth and predicted PETCO_2_ time courses are shown in upper and lower rows, with the corresponding correlation-coefficient histograms showing the comparability of the maps. The slice positions are shown by the yellow lines on the sagittal image in the upper-left corner.

Figure 8 demonstrates the application of the FCN-4L-predicted dynamic PETCO_2_, which have established correlation with the resting-state fMRI signal. We show that the PETCO_2_-fMRI correlation maps for the ground-truth and predicted PETCO_2_ are highly similar in all scan sessions (Cases 1, 2 and 3) and subjects (Datasets 1 and 2). This preliminary demonstration suggests promise in using the model-predicted PETCO_2_ for fMRI applications.

## 4. Discussion

As a proof-of-concept study, we demonstrated that it is feasible to use an FCN to predict dynamic CO_2_ from respiration variations. Furthermore, the performance of the FCN surpasses that of regression and convolution-based methods. Note that the results only pertain to dynamic patterns in CO_2_, not to absolute CO_2_, which cannot be predicted from non-quantitative respiration traces alone. Nonetheless, possible applications range from improving the feasibility of breath-holding based fMRI studies (Murphy et al., 2013) that lack CO_2_ recordings, to the use of the CO_2_-O_2_ exchange ratio for vascular reactivity mapping (Chan et al., 2020). These applications do not require quantitative values of CO_2_ and PETCO_2_.

### 4.1. Machine learning in physiological signal processing

The use of machine learning and DL models is prevalent in physiological signal data such as electromyogram (EMG), electroencephalogram (EEG), electrocardiogram (ECG), and electrooculogram (EOG) (Rim et al., 2020). It has been continuously observed that DL models perform better than other, classical machine learning models. Rim et al. conducted a review of 147 studies using DL in EMG, ECG, EEG, EOG and their combinations (Rim et al., 2020), and concluded that most were in the domain of classification, feature-extraction and data compression, wherein CNN, RNN, CNN+RNN models were most commonly used. The studies were divided into 3 categories. The first category exploits machine-learning models to extract features followed by DNN as a classifier to boost the accuracy of classification by obtaining useful features from raw data. The second involves DL as a feature extractor and traditional machine learning as a classifier to reduce hand-crafted labeling of the dataset. The third strategy uses an end-to-end DL pipeline to train raw data and receive the final output to build a robust model for the above-mentioned tasks. Due to the absence of a comparative study involving all 3 methods (Rim et al., 2020), we could not assess the best strategy. Our pipeline is positioned between the second and third categories, as we used an end-to-end DNN to estimate CO_2_ as an intermediate step, followed by a post-processing step to obtain the final PETCO_2_ waveform.

### 4.2. Utility and current status of using RVT for generating PETCO_2_

As RVTRRF is correlated with PETCO_2_, there is a potential of training a convolutional neural network between RVT and PETCO_2_, which might perform better than a single convolution operation using RRF. This approach aims to find a neural network architecture which could replace the need of RRF. We experimented with different types of neural networks trained to predict PETCO_2_ from RVT, but none performed adequately. Therefore, we concluded that it is more feasible to design a neural network to associate respiration and CO_2_, and predict PETCO_2_ from CO_2_. This may be due to the fact that the latter exploits the evident breathing pattern between respiration patterns and CO_2_ and performs well even with limited recording lengths. Conversely, in the former approach, the temporal resolution of RVT is fundamentally constrained to the observed breath durations, and the peak detection algorithm can often miss deep breaths (Power et al., 2020).

As a potential alternative metric of respiratory variability, the windowed respiratory variance (RV), computed as the standard deviation of the respiratory signal over sliding windows of 6 s (Chang et al., 2009), is more robust against noise than RVT as it excludes the influence of breath-cycle duration term. This may however render RV less physiologically related to CO_2_. Moreover, the RRF for RV has not been determined (Birn et al., 2008), leading us to exclude the use of RV in this proof-of-principle study. Another potential influence on CO_2_ prediction may be the presence of hardware/software filters on the raw recordings. The Biopac system provided software filters to exclude MRI noise (periodicity < 100 ms) while preserving higher physiological frequencies, and it is conceivable that in cases where such frequencies are inadvertently removed from the raw respiratory traces, the ability to predict CO_2_ fluctuations may be disadvantaged.

### 4.3. Other DL architectures

As mentioned previously, a 1D U-net with skip connections had previously been used for translating fMRI data to respiratory-volume data [30]. Skip connections as used in the U-net could be implemented in this study, but as the study is more focused on establishing proof of concept, such complications were avoided in our implementation of FCNs.

There are recently developed alternative network architectures that may also suit our problem. For instance, unpaired and paired image-to-image translation has been accomplished by generative adversarial networks (GANs) such as Pix2Pix (Isola et al., 2017) and CycleGAN (Zhu et al., 2017). The translation task is analogous to the task of transforming the respiration-belt data to the CO_2_ waveform is analogous. A simple GAN consists of two sub-models, a generator to obtain synthetic samples, and a discriminator to predict the value of the provided sample. The discriminator network in GANs is similar to the explicit loss function used in traditional DL models. In our case, adversarial training would mean that instead of using MSE or weighted MSE loss functions to determine the best CO_2_ prediction, another network would distinguish between them. Given that our use case is much simpler, this approach might not add value while incurring higher computational costs and overfitting.

Another alternative are RNNs, such as the long-short term memory (LSTM) (Greff et al., 2017) and gated recurrent unit (GRU) (Zhao et al., 2016) networks, which are widely used in signal processing. At first glance, RNNs seemed a natural choice, but unfortunately, performance was poor (data not shown) for the LSTM. In our implementation, the initial 5-s respiration-signal segment was fed into the LSTM block which would predict the corresponding segment of CO_2_ and the hidden state. These outputs along with the next 5-s segment of respiration data were used as the inputs for the next iteration, with the intention that irregularities in breathing would be stored in the network's memory and would help in prediction. Moreover, the 5-s length was comparable to the duration of one breath. Unfortunately, due to the short input-lengths coupled with the limited durations of respiration recordings, the concatenated output lacked the smooth transitions between consecutive chunks (i.e. edge effects were apparent in each 5-s block, similar to observed in training method 1), which are required for accurately predicting a slow-varying signal like PETCO_2_. Thus, we concluded that time-series to time-series translation using RNNs was not feasible unless much longer respiratory and CO_2_ recordings were available.

### 4.4. Limitations

Data quality can be a chief limitation in our approach, and we recommend careful quality assurance as indicated in this work. Another potential limitation is the way in which the test and training data are determined by splitting the full data set; the use of k-fold cross-validation reduces such bias. Peak detection accuracy, which determine the quality of the source PETCO_2_ data, also needs careful quality assurance. Finally, our method does not attach quantitative values to the estimated PCO_2_ or PETCO_2_ (e.g., in units of mmHg). This is because the quantitative value of PETCO_2_ depends not only on respiratory patterns, but also on minute ventilation, tidal volume, fitness level, baseline CO_2_ storage, and so on (Rawat et al., 2021). Nonetheless, our breath-by-breath CO_2_ time series reflects patterns of change are sufficient for fMRI applications.

## 5. Conclusions

This study demonstrates the feasibility of predicting dynamic PETCO_2_ from respiration-belt recordings, thus, enabling broader incorporation of PETCO_2_ in rs-fMRI analysis. Following the successful application of 2D FCNs to image-to-image translation, we introduced 1D FCNs for 1D signal-to-signal translation. The FCN outperformed the analytic regression and convolution models. The study also evaluates the effect of FCN depth as well as the choice of loss function. A 4-layer FCN with weighted MSE performed best across all splits. The results across different deep neural network architectures serve as a literature for further research in signal processing and for the DL community.

## Data availability statement

The raw data supporting the conclusions of this article will be made available by the authors, without undue reservation, in accordance with institutional guidelines.

## Ethics statement

The studies involving human participants were reviewed and approved by Baycrest Research Ethics Board. The patients/participants provided their written informed consent to participate in this study.

## Author contributions

VA: the conception or design of the work, analysis, interpretation of data for the work, drafting the work or revising it critically for important intellectual content, and final approval of the version to be published. XZ: analysis, interpretation of data for the work, drafting the work and in revising it for important intellectual content, and final approval of the version to be published. JC: the conception or design of the work, interpretation of data for the work, drafting and revising it critically for important intellectual content, and final approval of the version to be published. All authors agree to be accountable for all aspects of the work in ensuring that questions related to the accuracy or integrity of any part of the work are appropriately investigated and resolved.
