# Supplementary material for: Generating dynamic carbon-dioxide traces from respiration-belt recordings: Feasibility using neural networks and application in functional magnetic resonance imaging
Source: Front Neuroimaging. 2023 Feb 16;2:1119539. doi: 10.3389/fnimg.2023.1119539 (PMC10406216; doi:10.3389/fnimg.2023.1119539)
Supplement: Supplementary file 1 [file Data_Sheet_1.docx]

***SUPPLEMENTARY MATERIALS***

_
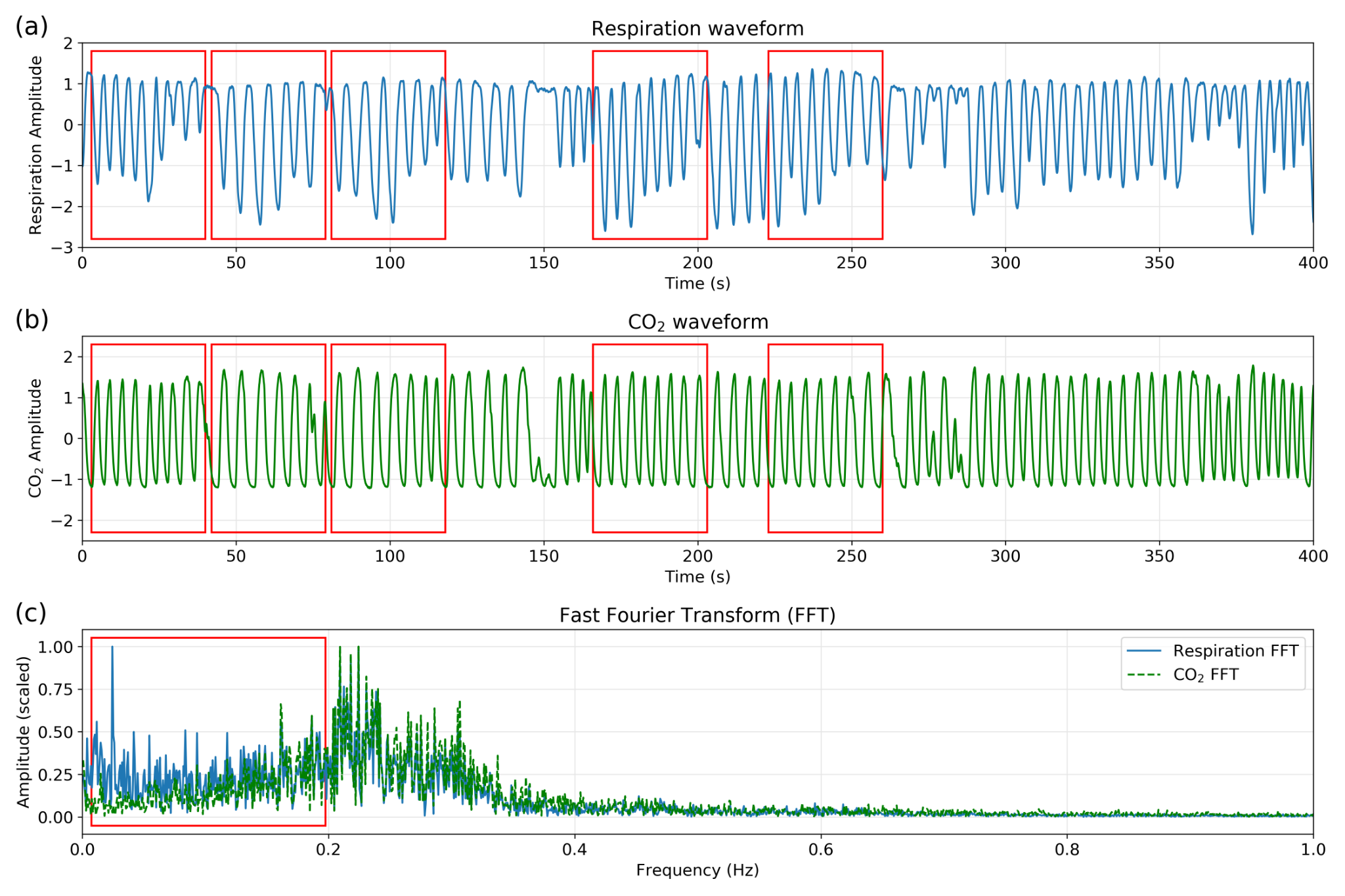
_

**Figure A1.** **Quality assurance: sample data with low-frequency noise**. (a) and (b) show a 400 seconds segment of the normalized respiration and CO_2_ recordings, respectively. In (c), the power spectra are shown. Red boxes superimposed on the plot are used to show the low frequency noise present in the data. Notice there is no low-frequency spectral mismatch in this clean data set.

_
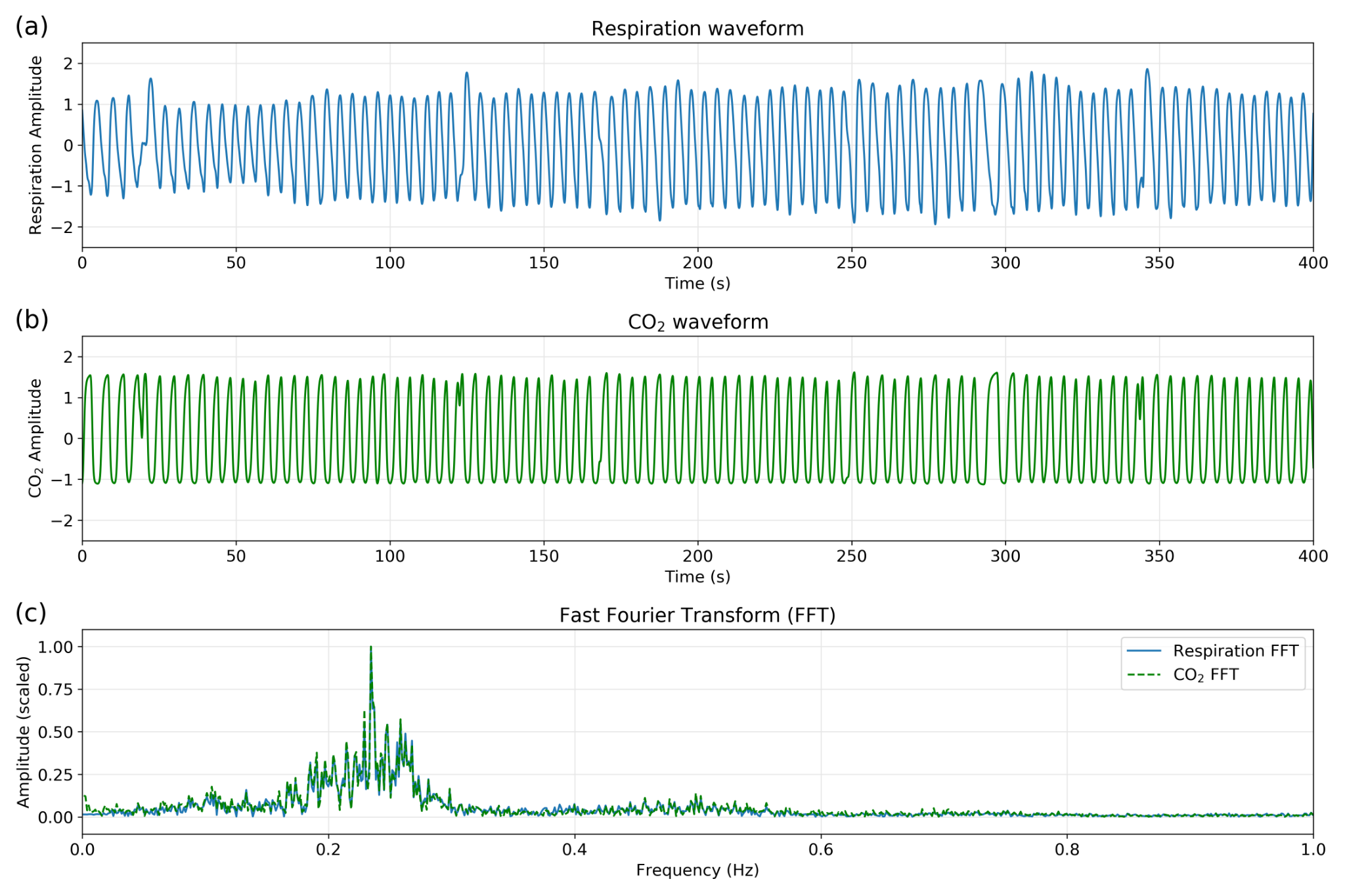
_

**Figure A2.** **Quality assurance: sample clean data.** Segment of a recording from our dataset. Similar to Figure 3, (a) and (b) correspond to normalized respiration and CO_2_ recordings respectively and (c) shows their frequency spectra.
